# The Application of PVDF-Based Piezoelectric Patches in Energy Harvesting from Tire Deformation

**DOI:** 10.3390/s22249995

**Published:** 2022-12-19

**Authors:** Kevin Nguyen, Matthew Bryant, In-Hyouk Song, Byoung Hee You, Seyedmeysam Khaleghian

**Affiliations:** 1 Department of Mechanical Engineering, University of Texas at Austin, Austin, TX 78712, USA; 2 Department of Mechanical Engineering, Montana State University, Bozeman, MT 58718, USA; 3 Department of Engineering Technology, Texas State University, San Marcos, TX 78666, USA

**Keywords:** energy harvesting, tire deformation, piezoelectric sensors, PVDF

## Abstract

The application of Polyvinylidene Fluoride or Polyvinylidene Difluoride (PVDF) in harvesting energy from tire deformation was investigated in this study. An instrumented tire with different sizes of PVDF-based piezoelectric patches and a tri-axial accelerometer attached to its inner liner was used for this purpose and was tested under different conditions on asphalt and concrete surfaces. The results demonstrated that on both pavement types, the generated voltage was directly proportional to the size of the harvester patches, the longitudinal velocity, and the normal load. Additionally, the generated voltage was inversely proportional to the tire inflation pressure. Moreover, the range of generated voltages was slightly higher on asphalt compared to the same testing conditions on the concrete surface. Based on the results, it was concluded that in addition to the potential role of the PVDF-based piezoelectric film in harvesting energy from tire deformation, they demonstrate great potential to be used as self-powered sensors to estimate the tire-road contact parameters.

## 1. Introduction

Increasing the number of onboard sensors in advanced and autonomous vehicles leads to the improved performance of different control algorithms in these vehicles. In turn, that improves the overall performance and safety of advanced vehicular transportation. In automobiles, tires are parts that need to be monitored closely since they are the only part of the vehicles that is in contact with the road and act as an interface between the vehicle control systems and the external environment; failure of the tires leads to the overall failure of the vehicles. Using a relatively new technology, “Intelligent Tires” (using different sensors attached to the tire inner-liner to monitor the tire-road interaction), the overall performance can be monitored continuously, and some of the tire contact parameters can be estimated [1].

Two major types of contact sensors are used for this purpose: accelerometers and piezoelectric strain sensors. Accelerometer-based studies use the acceleration signal (reflects the vibration of the tire’s inner liner) in time and frequency domains to monitor the tire condition [2,3], identify the road surface type [4], estimate the tire-road friction [5,6], and evaluate the road surface conditions (dry, wet, ice, or snow) [7]. Accelerometers can be integrated with different systems easily and can work over a wide range of temperatures; however, they are not compatible with large deformations [8].

Piezoelectric strain sensors are preferred to estimate the tire’s overall operational condition since they can directly measure tire deflection. Moreover, the strain signals are less noisy when compared to accelerometers; however, their sensitivity varies with temperature. Hence they require additional compensation circuits [9]. Piezoelectric strain sensors are widely used for tire health monitoring [10,11] and to estimate tire-road friction [12,13,14].

Although using sensors in different parts of vehicles, such as the tire’s inner liner in intelligent tire systems, improves the vehicle’s performance, there are still several hurdles associated with these systems that limit their applications. Increasing the number of sensors in modern vehicles will increase the length of wires, which further increases the vehicle’s weight and the need for space, reducing the vehicle’s reliability. Moreover, using the vehicle’s battery-powered sensors will increase the load on the vehicle’s main battery and decrease its lifetime. For sensors that use their own battery, a lower sampling frequency should be used in wireless data transmitters due to the limited availability of energy that reduces the drivers’ reaction time in emergency conditions [15]. Further, the labor cost of battery replacement is significant for these sensors. Therefore, designing an energy harvesting system that can power wireless data transmitters is critical for modern vehicles that use many sensors.

Tires experience abundant vibration and strain energy during vehicle operation; therefore, they can be used for energy harvesting to power wireless sensors. Various energy harvesting technologies have been developed to harvest energy from different parts of vehicles. Most vehicle-related energy harvesting technologies are based on vehicle vibration and/or exhaust heat [16,17]. Transductions are used in most tire energy harvesting studies, and some are introduced next.

Electromagnetic (EM) power generators were used in many studies to scavenge the mechanical vibration of tires [18,19]. Despite being cost-effective, integrating EM-based energy harvesters is difficult due to their large sizes, which limits their usage in harvesting energy from the tires. Microfiber piezoelectric composites [20,21,22] and nanogenerators (based on tribo-electric and Zinc Oxide (ZnO)) [23,24] were also used in several studies to harvest energy from the tire. Both are flexible in nature and provide reasonable power density. However, despite having a complex and expensive fabrication process (which increases the overall cost of the tires), their output power was not significant, and they were susceptible to the temperature, which makes them unsuitable for energy harvesting from the tires [21]. PZT, or lead zirconate titanate, is one of the world’s most widely used piezoelectric ceramic materials. However, the limited frequency range of the PZT resonance, which are not functional beyond a specific frequency range, and the need for tip masses, which increases the overall weight of the harvester system, make them inappropriate to be used for tire energy harvesting. Polyvinylidene Fluoride or Polyvinylidene Difluoride (PVDF) is another piezoelectric material that generates voltage under pressure or axial force [25]. PVDF is one of the most attractive materials widely used for mechanical energy harvesting applications [26]. Its features, such as a high piezoelectric coefficient, excellent stability, and high flexibility, make it suitable for use in different nanogenerators [27,28]. PVDF’s stable behavior under temperature changes, excellent chemical resistance, high flexibility, mechanical strength and toughness, relatively low stiffness (to not change the tire behavior), and ease of use and integration make it appropriate for energy harvesting in the tire’s harsh environment (high temperature and extreme deformation). Although PVDF-based piezoelectric materials have been used in many studies for applications like pressure sensors [29,30] and measuring flow velocity [31], their performance in energy harvesting from tire deformation under actual testing conditions has remained relatively unknown.

In order to study the effectiveness of PVDF in energy harvesting from tire deformations, an instrumented tire with different sizes of PVDF piezoelectric patches and a tri-axial accelerometer was prepared and tested under different conditions using an instrumented vehicle. Longitudinal velocity, normal load, tire inflation pressure, and pavement type were among the investigated testing conditions. The remainder of this paper is structured as follows. The experimental test setup used in this study is explained in Section 2, followed by the design of the experiment, which is explained in Section 3. Results and discussion are presented in Section 4, followed by conclusions in Section 5.

## 2. Experimental Test Setup

In order to study the application of PVDF piezoelectric materials in tire energy harvesting, an instrumented truck was used. Next, different parts of the test setup are explained.

### 2.1. Instrumented Tires

**Figure 1:**
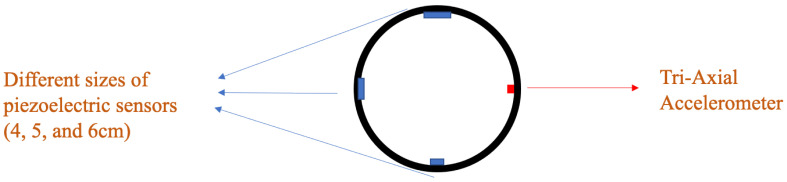
The arrangement of different sensors in the instrumented tire.

Two instrumented tires were prepared with three piezoelectric patches and a tri-axial accelerometer each. The schematic of the instrumented tire with embedded sensors is shown in Figure 1, with the location of different sensors.

#### 2.1.1. PVDF Piezoelectric Patches

**Figure 2:**
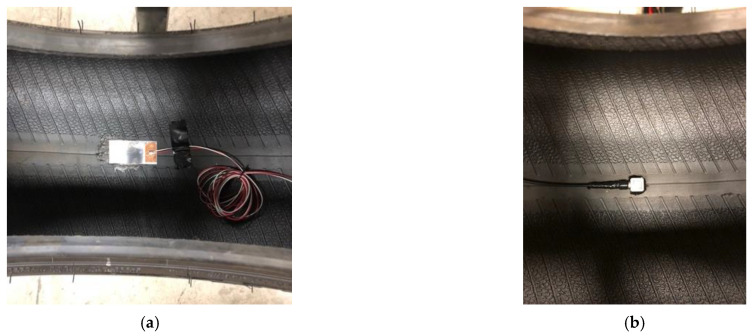
The sensors attached to the tire’s inner liner: (**a**) PVDF-based piezoelectric sensor, and (**b**) tri-axial accelerometer.

In order to study the effects of the piezoelectric patches’ size on the output voltage (considering that the curvature of the tire may bend and damage the patches), three PVDF-based piezoelectric patches with different lengths (4 cm, 5 cm, and 6 cm) and a width of 2 cm were cut from a 297 cm × 210 cm sheet of KUREHA KF Piezo film with a thickness of 200 μm and a piezoelectric constant d_31_ of 25 pc/N. The patches were attached to the tire’s inner liner using silicon-based adhesive. Two wires were then connected to the positive and negative sides of the piezoelectric patches using copper tape with a conductive adhesive; a sample piezoelectric patch used in this study is shown in Figure 2a. These lengths of piezoelectric patches were selected based on the common size of off-the-shelf piezoelectric patches used in other studies. The list of properties of a commercial PVDF-based piezoelectric material is presented in Table 1.

#### 2.1.2. Tri-Axial Accelerometer

In order to monitor the vibration of the contact patch area, a tri-axial accelerometer was also attached to the tire’s inner liner. In this study, the Dytran model 3053B, with a sensing range of ±500 g and sensitivity of −10 mv/g in x, y, and z directions, was used. The tri-axial accelerometer used in this study is shown in Figure 2b.

#### 2.1.3. Connectors on the Rim

**Figure 3:**
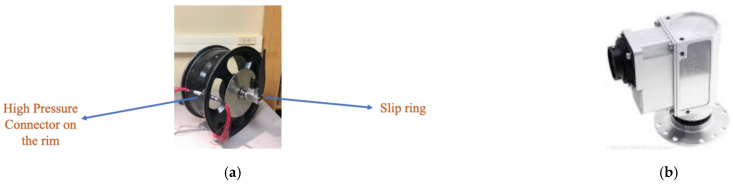
(**a**) High-pressure connectors (male and female) used in this study. (**b**) The waterproof slip ring used in this study.

Since the sensors used in this study are still wired, a hole was drilled into the rim, and a specialized high-pressure connector was placed on the rim. The rim with the connector is shown in Figure 3a. The sensor wires were connected to the inner side of the connector, and the wires from the outer side were connected to the slip ring, which is explained next.

#### 2.1.4. Slip Ring

Since the wires are rotating with the rim, a slip ring, which is an electromechanical device that allows the transmission of power and electrical signals from a stationary to a rotating structure, was used to connect the wires to the data collecting device. The slip ring is shown in Figure 3b. The SR10AW-E512-TX rev 2002 from Michigan scientific was used in this study. It is waterproof and has a built-in 512PPR encoder, so the wheel’s angular velocity was also measured.

#### 2.1.5. Other Vehicle Sensors

**Figure 4:**
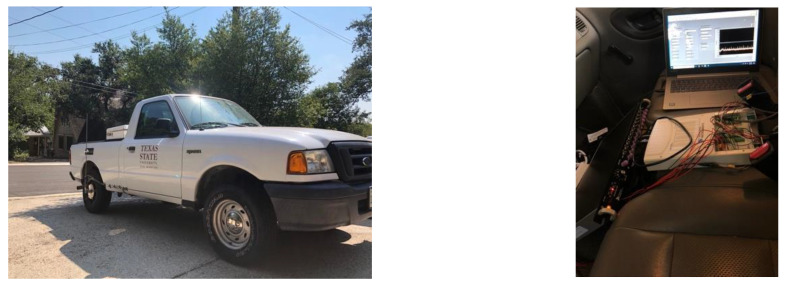
The instrumented truck used in this study.

In addition to the smart tires, the vehicle (2004 Ford Ranger) used in this study was instrumented with other sensors to capture its dynamics properly. A steering wheel measurement was used to measure the steering angle and steering velocity. Moreover, an Inertial Measurement Unit (IMU) was placed at the vehicle’s approximate Center of Gravity (CG) to measure the acceleration components and the roll, pitch, and yaw rate. The instrumented vehicle used in this study is shown in Figure 4.

#### 2.1.6. Data Collecting System

**Figure 5:**
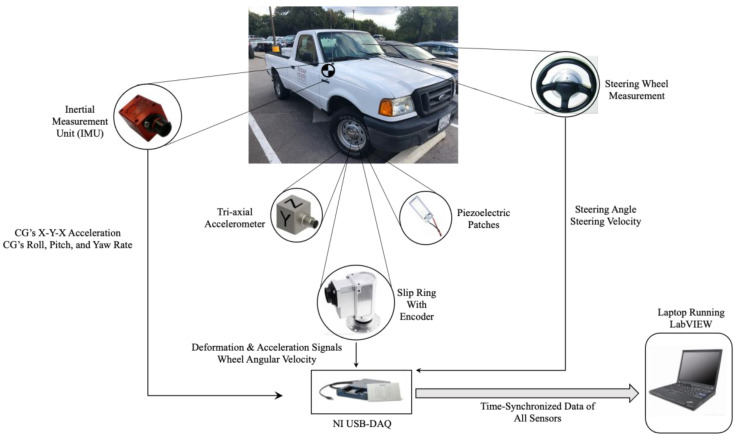
Schematic of the data collecting system used in the instrumented truck.

In order to collect the data of all sensors, a USB NI-DAQ with 32 differential channels and two counter inputs was used, and a data collecting routine was developed in LabVIEW that collected the time-synchronized data of all sensors with the desired sampling rate. In this study, a sampling frequency of 1000 Hz was used for all the experiments. The schematic of the instrumented truck with the data collecting system is shown in Figure 5.

In order to evaluate the performance of the PVDF-based piezoelectric patches in harvesting energy from tire deformation, an experiment was designed with different testing conditions and is explained next.

## 3. Experiments

In order to evaluate the performance and effectiveness of PVDF-base piezoelectric patches in harvesting energy from tire deformations under different testing conditions, an experiment was designed using the instrumented truck. The time-synchronized data of all sensors and harvesters were collected at four different longitudinal speeds: 15, 20, 25, and 30 mph (6.7, 8.9, 11.17, and 13.41 m/s respectively); two tire inflation pressures, nominal—35 psi (241.31 KPa) and below nominal—25 psi (172.37 KPa); with and without the presence of 240 lb (108.86 kg) of additional static weigh on the back of the truck; and on two different surfaces, asphalt and concrete. The sampling frequency of 1000 Hz was used in all cases since it is practical in the real world (does not take too much capacity), and the overall shape of the signal was still intact.

## 4. Results and Discussion

**Figure 6:**
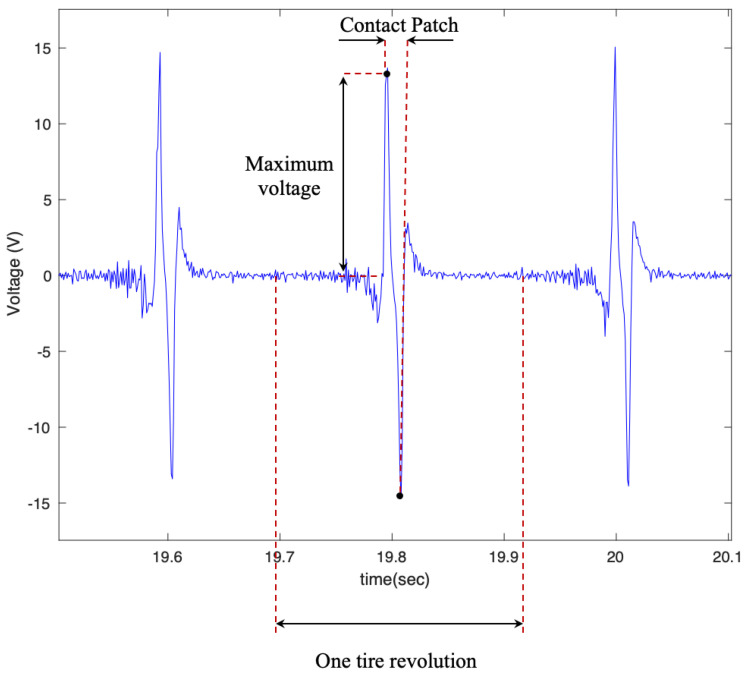
The overall shape of the output signal, from the 6 cm piezo patch, with a tire pressure of 35 psi and a longitudinal velocity of 25 mph.

The overall shape of the output voltage is shown in Figure 6, which matches the expected output of the PVDF patches [32]. Considering the relationship between the output of the flex sensors and the PVDF, the time difference between two peaks, as shown in Figure 6, multiplied by the longitudinal velocity of the vehicle, represent the contact patch length.

**Figure 7:**
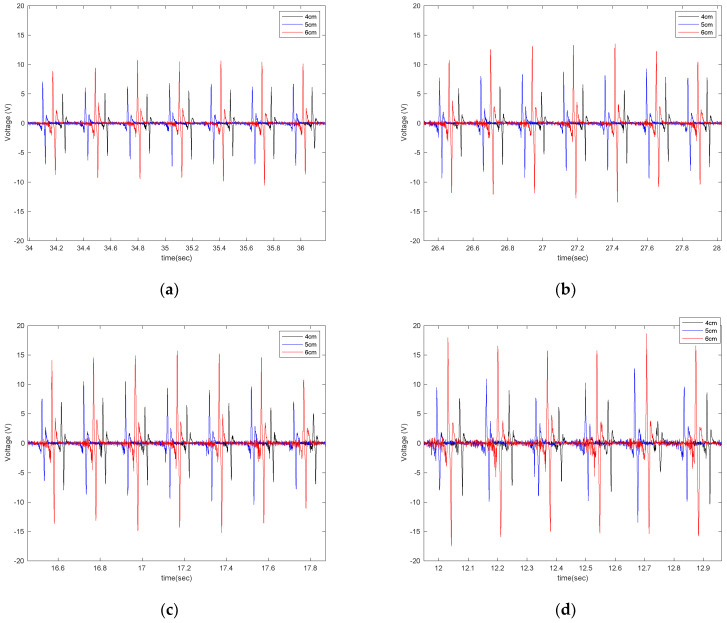
The effect of sensor length on the output signal at the longitudinal velocity of (**a**) 15 mph (**b**) 20 mph, (**c**) 25 mph, and (**d**) 30 mph, all from a tire with an inflation pressure of 35 psi and no additional normal load.

In order to evaluate the effect of piezoelectric size on the output voltage, three different sizes of PVDF-based piezoelectric patches (4 cm, 5 cm, 6 cm) were attached to the tire’s inner liner and tested under the instrumented truck. These specific sizes were selected based on the common size of off-the-shelf piezoelectric sensors used in different studies. The output signals are shown in Figure 7 for different longitudinal speeds, at a tire inflation pressure of 35 psi, and with no additional load on the back of the truck while on an asphalt surface.

**Figure 8:**
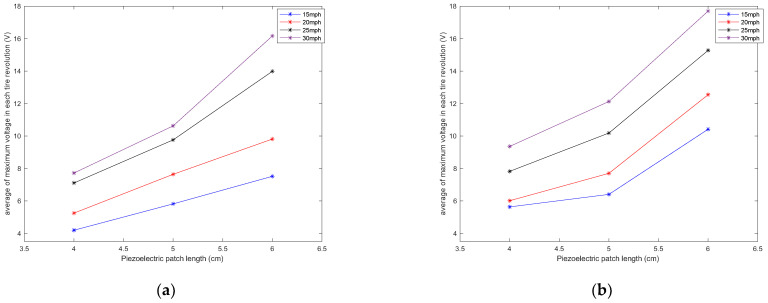
Sensitivity analysis of the average of maximum voltage in each tire revolution to the length of the piezoelectric patches for a tire pressure of (**a**) 35 psi and (**b**) 25 psi, both without the presence of additional normal load.

It is observed that the generated voltage is proportional to the length of the piezoelectric patches in all longitudinal velocities. As the length of the piezoelectric patches increases, the generated voltage range also increases. This was expected since the same deflection was applied on all of the piezoelectric patches, as they are all attached to the same tire and experience the same amount of deformation. A larger sensor patch length means more energy harvester material, leading to the generation of a larger voltage range. This also can be seen in Figure 8, in which the average values of maximum voltage (shown in Figure 6) for each tire revolution were plotted versus the PVDF-based piezoelectric patch length for different longitudinal velocities. The maximum generated voltage in each tire revolution was averaged over an interval of at least 20 tire revolutions. It is observed that for all the tested longitudinal velocities and tire pressures, the maximum voltage is proportional to the piezoelectric patch length; however, the relationship is nonlinear.

**Figure 9:**
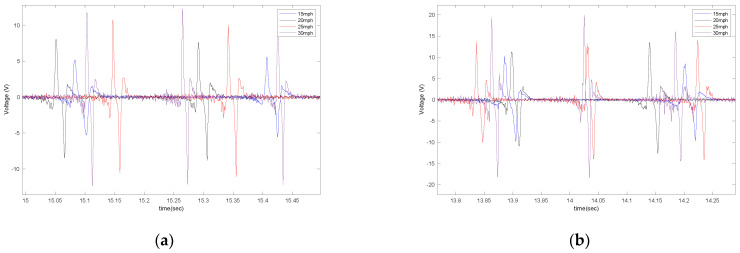
The effect of longitudinal velocity on the output signal of the (**a**) 5 cm patch and (**b**) 6 cm patch, at 35 psi with no additional load.

In Figure 9, the effect of longitudinal velocity on the output voltage is presented for two different sizes of sensor patches (5 cm and 6 cm), with a tire pressure of 35 psi and no additional load while on asphalt.

**Figure 10:**
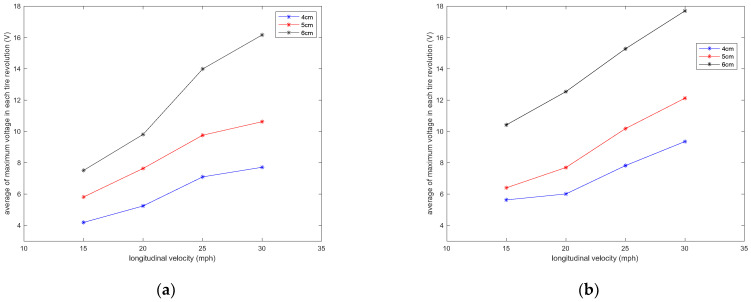
Sensitivity analysis of the average of maximum voltage in each tire revolution to the longitudinal velocity in tire pressures of (**a**) 35 psi and (**b**) 25 psi, both without the presence of additional normal load.

It is observed that velocity also has a significant effect on the amount of generated voltage. A higher longitudinal velocity led to a larger range in the generated voltage. The sensitivity analysis of the averaged maximum voltage for each tire revolution to the longitudinal velocity is presented in Figure 10 for different lengths of piezoelectric patches and tire pressures. It is observed that the relationship between the maximum generated voltage and longitudinal velocity is almost linear; however, the rate of the maximum voltage-change over velocity increases as the size of PVDF-based piezoelectric patches increases.

**Figure 11:**
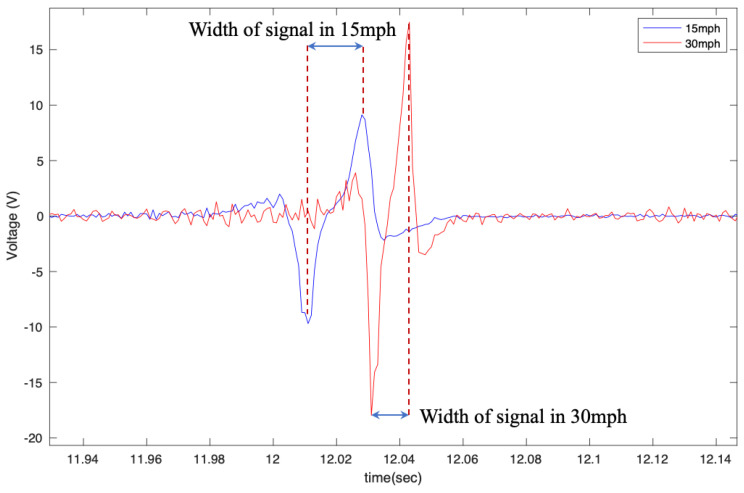
Width of the output voltage in different longitudinal velocities from the 6 cm patch at 35 psi with no additional load condition.

Moreover, the width of the signal decreases as the velocity increases, which was also expected since the width of the signal was correlated to the amount of time that the sensor was in the contact patch. This is shown in Figure 11. This characteristic of the signal can be used to estimate the contact patch length and the normal load that is applied to the tire since it is correlated to the contact patch length.

**Figure 12:**
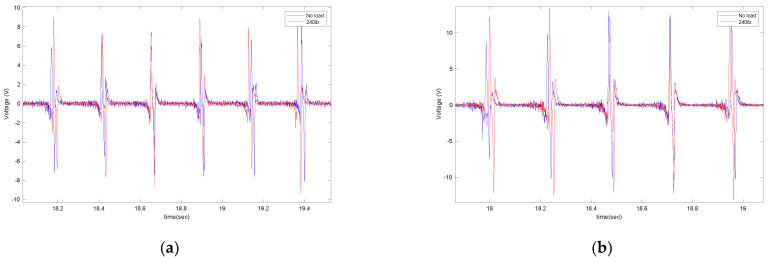
The effect of external load on the output voltage for (**a**) 4 cm piezo patches and (**b**) 5 cm piezo patches, both at a longitudinal velocity of 20 mph and 35 psi.

A static weight of 240lb was placed on the back of the truck to investigate the effect of normal load on the output signal. The generated signal was compared to the case without additional external load; the results shown in Figure 12 are for two different lengths of the piezoelectric patches (4 cm and 5 cm) on asphalt.

**Figure 13:**
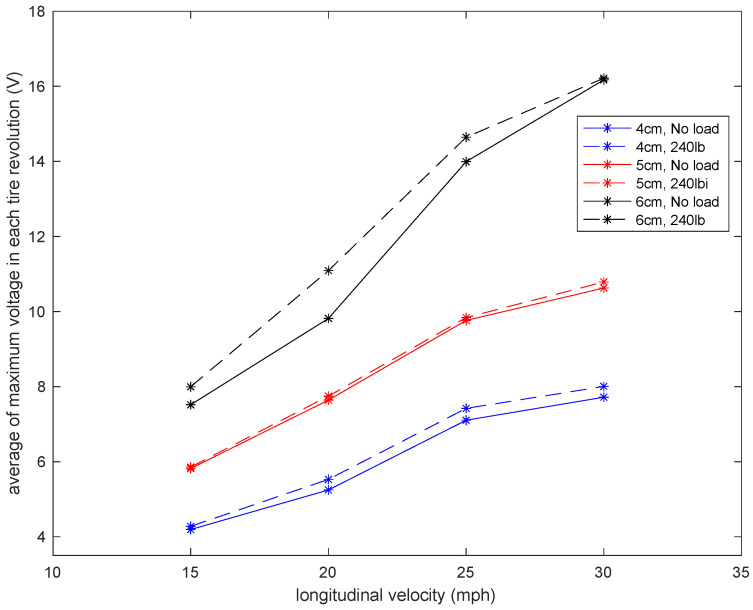
Sensitivity analysis of the average maximum voltage in each tire revolution to the longitudinal velocity in a tire pressure of 35 psi with and without the presence of the additional load.

It is observed that the generated voltage slightly increases when the normal load increases. This was also expected. Once the normal load increased, the deflection of the tire in the contact patch area (where the sensor is attached) increased. Since the generated voltage was directly correlated to the deflection, a slightly higher voltage was generated in a higher normal load. However, the voltage difference is not significant. The same trend holds for the averaged maximum value of the generated signal, presented in Figure 13. It is observed that for all velocities and piezoelectric patch lengths, the maximum generated voltage increases as the normal load on the tire increases. However, the increase in the maximum values of generated voltage due to the normal load increase is more significant in the 6 cm piezoelectric patch than in the smaller patches.

**Figure 14:**
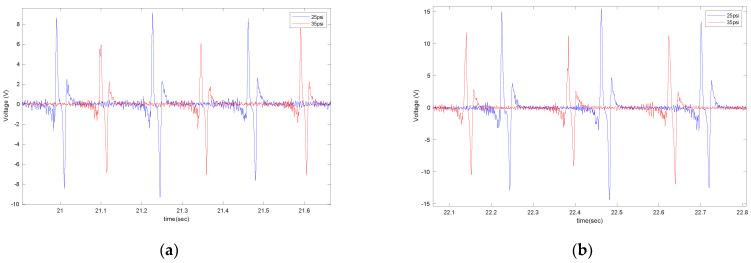
The effect of tire pressure on the output voltage for (**a**) 4 cm piezo patches and (**b**) 6 cm piezo patches, both at a longitudinal velocity of 20 mph, with no additional load, and on asphalt.

The effect of tire pressure on the output signal is shown in Figure 14 for two different sensor sizes (4 cm and 6 cm), at a velocity of 20 mph and with no additional load.

**Figure 15:**
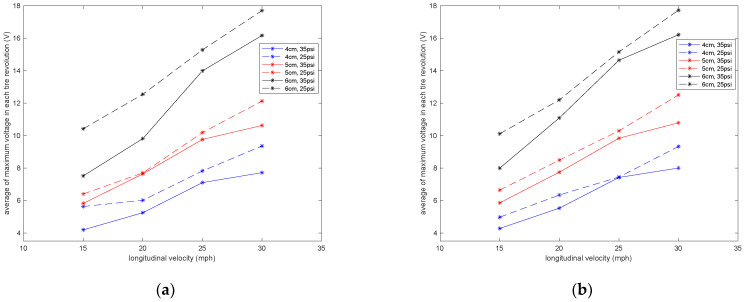
Sensitivity analysis of the average maximum voltage in each tire revolution to the longitudinal velocity in tire pressure (**a**) without presence of the additional load and (**b**) with presence of the additional load.

Once the tire pressure dropped, the amount of tire deflection in the contact patch area increased, which led to the generation of a higher voltage in all lengths of the PVDF-based piezoelectric patches. The sensitivity analysis of the averaged maximum of generated voltage in each tire revolution to the longitudinal velocity in tire pressure with and without the additional load is shown in Figure 15. It is observed that the maximum generated signal increases with the tire pressure drop in all velocities and piezoelectric patch lengths, and the relationship between the maximum generated voltage and the longitudinal velocity is almost linear, especially for the low tire inflation-pressure case.

**Figure 16:**
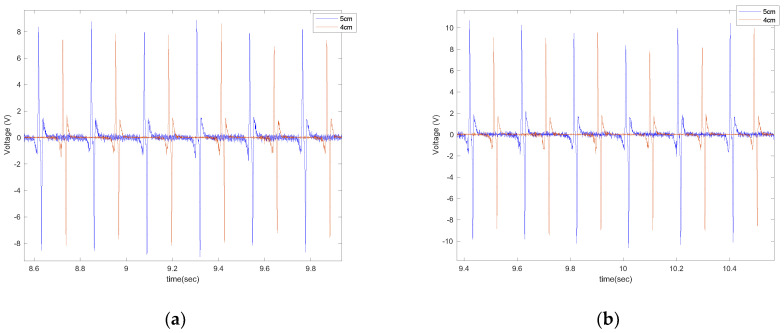
The effect of length on the generated voltage: (**a**) 20 mph and (**b**) 25 mph, both for the tire with 35 psi and with no additional load condition while on concrete.

**Figure 17:**
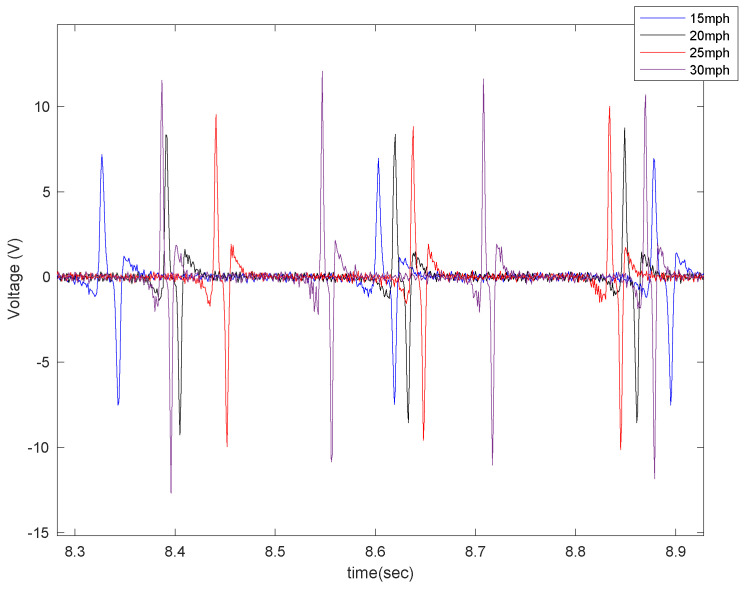
The effect of longitudinal velocity on the generated voltage in the 5 cm piezo patch under 35 psi of tire pressure and with no additional load while on concrete.

**Figure 18:**
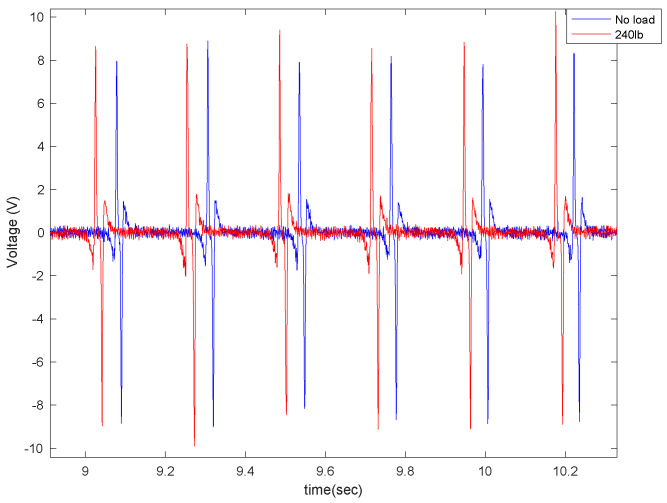
The effect of tire normal load on the generated voltage for the 5 cm patch at a velocity of 20 mph with a tire pressure of 35 psi while on concrete.

**Figure 19:**
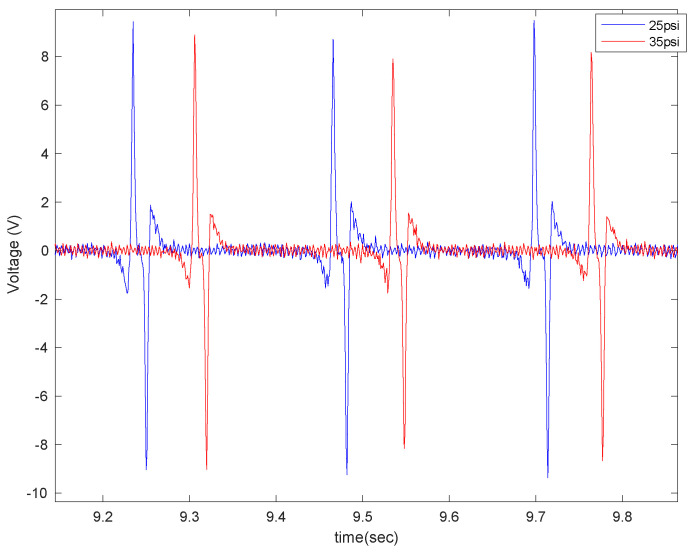
The effect of tire pressure on the generated voltage for the 5 cm patch at a velocity of 20 mph and with no additional load while on concrete.

Next, the experiment was repeated on concrete pavement. The results are shown in Figure 16, Figure 17, Figure 18 and Figure 19 for the effect of piezo patch length, longitudinal velocity, normal load, and tire pressure on the generated voltage, respectively.

**Figure 20:**
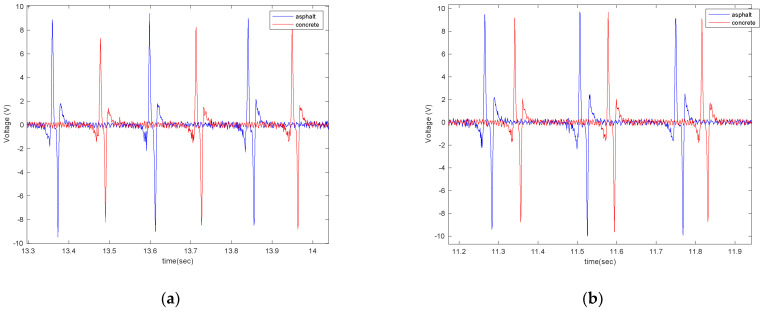
The generated voltage from the 5 cm piezoelectric patch for a tire pressure of (**a**) 35 psi and (**b**) 25 psi, both at a longitudinal velocity of 20 mph and with no additional load.

**Figure 21:**
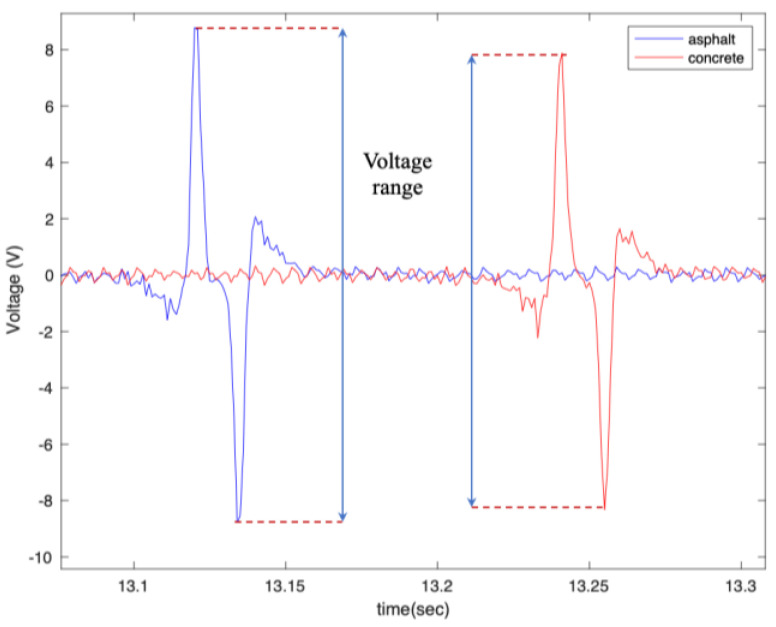
The effect of surface type on the generated signal from the 5 cm piezoelectric patch at a longitudinal velocity of 20 mph, tire pressure of 35 psi, and with no additional load condition.

Similar to what was seen on the asphalt pavement, it is observed that the amount of generated voltage on the concrete pavement is correlated to the length of the harvester patch, the longitudinal velocity, the normal load, and tire pressure. It is directly proportional to the length of the patches, longitudinal velocity, and normal load and inversely proportional to the tire pressure. However, like asphalt, the effect of the normal load on the generated voltage was not significant. Next, the generated voltages on the asphalt and concrete surfaces are compared in Figure 20 and Figure 21.

As shown in Figure 20 and Figure 21, the generated voltage range on asphalt is slightly larger than on concrete. However, this significantly depends on the macro texture of the tested surfaces, and the results might be different for asphalt and concrete surfaces with different macro textures than those tested. Further studies are needed to draw a firm conclusion regarding the use of PVDF-based piezoelectric patches for pavement type identification. However, the results are promising for using the piezoelectric patches not only for energy harvesting but also for tire-road contact parameter estimation.

## 5. Conclusions

The possibility of harvesting energy from tire deformations was investigated in this study. PVDF-based piezoelectric patches were identified as an appropriate potential energy harvester for use inside the tire (harsh environment, high temperature, and extreme deformation) due to their high flexibility, low price, durability in a high-temperature environment, high frequency, and ease of integration. Three different sizes of PVDF-based piezoelectric patches (4 cm, 5 cm, and 6 cm, all with a width of 2 cm) were cut from a sheet of KUHERA KF Piezo film with a thickness of 200 μm. They were attached to the tire’s inner liner and tested under different conditions (different velocities, tire pressures, and with and without the presence of an external normal load) using an instrumented vehicle (Ford Ranger 2004) on two different pavement types. It was concluded that on both asphalt and concrete surfaces, the output voltage of the PVDF-based piezoelectric film is proportional to the size of patches, the longitudinal velocity, and the normal load. Additionally, the generated voltage is inversely proportional to the tire inflation pressure. The same trend holds for the relationship of the averaged maximum voltage in each tire revolution and those factors; it is proportional to the length of PVDF-based piezoelectric patches, the longitudinal velocity, and the normal load and is inversely proportional to the tire inflation pressure, with a relatively linear relationship in almost all cases. Moreover, the range of the generated voltage was higher on asphalt than on the concrete surface. However, a more comprehensive study is required to make a solid conclusion since the generated voltage mainly depends on tire deflection due to the pavement’s macro-texture, which may differ for various asphalt and concrete surfaces. Future works include the design and fabrication of an efficient energy harvesting circuit to harvest energy from tire deformation, the use of the PVDF-based piezoelectric patches in modified and worn-out tires, and the evaluation of their effectiveness in detecting any change in the tire structure.

## Figures and Tables

**Table 1:** Properties of the commercial PVDF-based piezoelectric [15].

Property	Unit	Value
Density	ρ 10^3^ kg/m^3^	1.78
Dielectric constant	ε *_r_*	13
Piezoelectric constant	*d*_31_ pc/N	25
	*e*_31_ mc/m^2^	75
	*g*_31_ mVm/N	220
Elongation	MD	% (at Break): 20–30
	TD	% (at Yield): 5–7

## Data Availability

Not applicable.
